# Pertuzumab Charge Variant Analysis and Complementarity-Determining Region Stability Assessment to Deamidation

**DOI:** 10.1021/acs.analchem.2c03275

**Published:** 2023-02-16

**Authors:** Baubek Spanov, Oladapo Olaleye, Tomés Mesurado, Natalia Govorukhina, Alois Jungbauer, Nico C. van de Merbel, Nico Lingg, Rainer Bischoff

**Affiliations:** † Department of Analytical Biochemistry, Groningen Research Institute of Pharmacy, University of Groningen, A Deusinglaan 1, 9713 AV Groningen, The Netherlands; ‡ Department of Biotechnology, Institute of Bioprocess Science and Engineering, University of Natural Resources and Life Sciences, Vienna, Muthgasse 18, Vienna 1190, Austria; § Bioanalytical Laboratory, ICON, Amerikaweg 18, 9407 TK Assen, The Netherlands

## Abstract

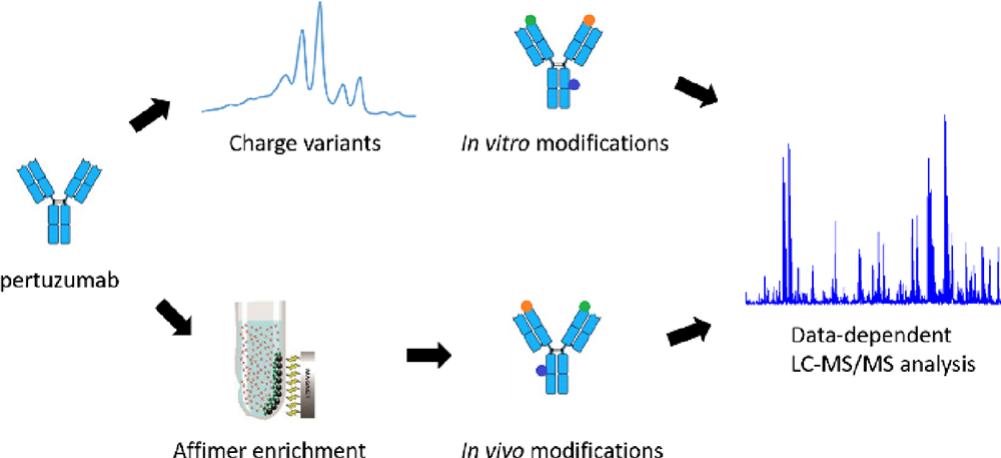

Pertuzumab is a monoclonal antibody used for the treatment of HER2-positive breast cancer in combination with trastuzumab. Charge variants of trastuzumab have been extensively described in the literature; however, little is known about the charge heterogeneity of pertuzumab. Here, changes in the ion-exchange profile of pertuzumab were evaluated by pH gradient cation-exchange chromatography after stressing it for up to 3 weeks at physiological and elevated pH and 37 °C. Isolated charge variants arising under stress conditions were characterized by peptide mapping. The results of peptide mapping showed that deamidation in the Fc domain and N-terminal pyroglutamate formation in the heavy chain are the main contributors to charge heterogeneity. The heavy chain CDR2, which is the only CDR containing asparagine residues, was quite resistant to deamidation under stress conditions according to peptide mapping results. Using surface plasmon resonance, it was shown that the affinity of pertuzumab for the HER2 target receptor does not change under stress conditions. Peptide mapping analysis of clinical samples showed an average of 2–3% deamidation in the heavy chain CDR2, 20–25% deamidation in the Fc domain, and 10–15% N-terminal pyroglutamate formation in the heavy chain. These findings suggest that *in vitro* stress studies are able to predict *in vivo* modifications.

## Introduction

Pertuzumab is a humanized monoclonal antibody (mAb) that targets the extracellular dimerization domain of the human epidermal growth factor receptor 2 (HER2). Pertuzumab is used in combination with either trastuzumab or ado-trastuzumab emtansine for the neoadjuvant treatment of metastatic HER2-positive breast cancer.^1−4^ Having a similar mechanism of action as trastuzumab, pertuzumab selectively binds subdomain II of the HER2 extracellular domain (ECD). Due to its open conformation, subdomain II of the HER2 ECD tends to form ligand-free homo- and/or heterodimers with other receptors from the HER family (HER1/EGFR, HER3, and HER4). Stronger mitogenic responses of HER heterodimers compared to HER homodimers have been reported earlier.^1,5^ This shows the importance of pertuzumab treatment in inhibiting HER2 dimerization and consequently tumor cell proliferation. Pertuzumab, like other mAbs, can also induce antibody-dependent cellular cytotoxicity and complement-dependent cytotoxicity, which are important functional mechanisms for anticancer efficacy.^6^ Dual anti-HER2 therapy with the combination of trastuzumab and pertuzumab has been reported to improve progression-free survival in the treatment of HER2-positive metastatic breast cancer compared to trastuzumab monotherapy.^2^

Charge heterogeneity, i.e., the occurrence of multiple proteoforms with different charge profiles, is common for mAbs and is often regarded as a critical quality attribute (CQA) for the manufacturing of pharmaceutical products.^7^ Charge variant analysis is one of the tools to monitor the lot-to-lot consistency of a product during the manufacturing process. Usually, charge variants of therapeutic mAbs are studied in detail since they can impact the safety and efficacy of the drug. Semi-preparative fractionation of the variants and subsequent characterization using various biophysical methods are the current state of the art.^8^ Charge variants can form during manufacturing, storage, and after administration through *in vivo* biotransformation. The charge variants generated along the life cycle of the mAb do not necessarily have to be the same, i.e., different heterogeneity might arise during manufacturing and *in vivo* biotransformation. Asparagine deamidation is the most common modification among others that contributes to charge heterogeneity because it converts a neutral amino acid (asparagine (Asn)) into a negatively charged one (aspartate (Asp) or isoaspartate (isoAsp)). When deamidation occurs in the complementarity-determining regions (CDRs) of antibodies, it may affect the affinity of antigen binding. Charge variants of trastuzumab have been extensively characterized in the literature and the sources of charge heterogeneity identified.^9−11^ However, little is known about charge variants of pertuzumab and notably the effect of deamidation on HER2 binding.

Charge heterogeneity of mAbs can be assessed using separation techniques such as isoelectric focusing and ion-exchange chromatography.^12^ The latter is often used for detailed characterization and functional assessments, because it allows the isolation of charge variants under native conditions.^10,13,14^ In this study, we followed the change in charge heterogeneity of pertuzumab by cation-exchange chromatography after stressing it at 37 °C for up to 3 weeks at physiological pH (PBS, pH 7.4) and basic pH (HEPES, pH 8.5). The basic pH was chosen here to help induce possible modifications such as deamidations. Charge variants of pertuzumab were characterized by peptide mapping after fractionation by cation-exchange chromatography. Peptide mapping is a widely used approach to determine modification sites of mAbs after enzymatic digestion.^10,11,14^

Pertuzumab (Perjeta) is a blockbuster antibody and its marketing exclusivity is expiring in May 2023 in Europe and in June 2024 in the United States.^15^ Several biopharmaceutical companies have started developing pertuzumab biosimilars.^16^ It is thus of great importance to understand the sources of charge heterogeneity and to identify CQAs of this therapeutic antibody. Pertuzumab has three asparagine residues in CDR2 of the heavy chain, namely, N52, N54, and N61, which is the only CDR containing asparagine residues (Figure S1). A study by Lu et al. reported that CDR2 of the heavy chain and CDR1 of the light chain in mAbs are most prone to deamidation.^17^ According to Vajdos et al., positions N52 and N54 in the CDR2 of pertuzumab are important for antigen binding.^18^ We, therefore, wanted to assess the susceptibility of CDR2 in the heavy chain of pertuzumab to deamidation after stressing at pH 7.4 and 8.5 and 37 °C. The susceptibility of the heavy chain CDR2 to deamidation was evaluated by peptide mapping of stressed pertuzumab. The changes in affinity to HER2 ECD of stressed pertuzumab were evaluated by surface plasmon resonance (SPR), which is considered the standard method to determine binding kinetics and affinities of antibody–antigen interactions.^19,20^

## Experimental Section

### Materials and Reagents

Pertuzumab (Drug Bank accession number: DB06366) (Perjeta, Lot H0319H03) was obtained from Roche. Human HER2/ErbB2 protein (His Tag protein, extracellular domain Thr23–Thr652; cat # HE2-H5225) was purchased from Acrobiosystems. C-terminally arginine-^13^C_6_-^15^N_4_-labeled GLEWVADVNPNSGGSIYNQR* peptide was purchased from JPT Peptide Technologies. Trypsin/Lys-C Mix, Mass Spec Grade (cat # V5073), was acquired from Promega. Difluoroacetic acid (DFA; cat # 162120025) was obtained from Acros Organics. dl-Dithiothreitol (DTT; cat # D0632), sodium deoxycholate (SDC; cat # 30970), and 4-(2-hydroxyethyl)piperazine-1-ethanesulfonic acid (HEPES; cat # H4034) were purchased from Sigma-Aldrich.

### Forced Degradation of Pertuzumab

Pertuzumab at a concentration of 2 mg/mL was stressed in PBS (pH 7.4) or HEPES (pH 8.5) buffers for up to 3 weeks at 37 °C. Stressed samples were collected at 1 week intervals and further analyzed by cation-exchange chromatography and LC–MS/MS peptide mapping.

### Charge Variant Separation by Cation-Exchange Chromatography

Charge variant analysis of pertuzumab was performed by pH gradient separation on a strong cation-exchange MabPac SCX-10 (4 × 250, 5 μm, Thermo Fisher Scientific) column, as described previously for trastuzumab.^10,21^ Buffer A had a pH of 8 and buffer B had a pH of 10.5. A gradient of 0 to 60% B in 63 min was applied. The flow rate was set to 0.5 mL/min. The UV absorption was measured at 280 nm. Fractions from the cation-exchange column, corresponding to the most abundant charge variants of pertuzumab, were collected based on their retention times.

### LC–MS/MS Peptide Mapping

Stressed pertuzumab samples and samples from fraction collection were denatured in the presence of 0.3% SDC in 50 mM HEPES (pH 7) and reduced with 10 mM DTT for 30 min at 60 °C for LC–MS/MS peptide mapping. Proteins were digested with a Trypsin/Lys-C Mix for 6 h at 37 °C at a protein-to-enzyme ratio of 30:1. SDC precipitation was performed by adding DFA to a final concentration of 0.4% and centrifugation for 10 min at 10000 rpm. Peptides generated after enzymatic digestion were separated on a μPAC capLC RP C18 column (5 μm pillar diameter, 50 cm bed length, PharmaFluidics). Mobile phase A consisted of 0.05% DFA in water, and mobile phase B was 0.05% DFA in acetonitrile (AcN). The gradient changed from 3 to 30% B in 80 min at a flow rate of 5 μL/min and a column temperature of 40 °C. The autosampler temperature was set to 8 °C. For a detailed description of the experiment, see page 3 of the Supporting Information.

### Intact Mass Measurement of Charge Variants by Reversed-Phase LC–MS

Intact mass measurements of charge variants fractionated by cation-exchange chromatography were performed on a Maxis Plus QTOF mass spectrometer (Bruker Daltonics, Bremen, Germany) coupled to a Waters Acquity UPLC (Waters, Milford, USA), as described previously.^10^ For a detailed description of the experiment, see page 3 of the Supporting Information.

### SPR

Protein–protein interactions were measured on a Biacore T200 system (Cytiva, Uppsala, Sweden) with a Series S Protein A sensor chip (Cytiva, Uppsala, Sweden). Pertuzumab was immobilized on the protein A chip to an immobilization level of 145 RU. Phosphate-buffered saline with 0.05% Tween 20 was used as the binding buffer. Single-cycle kinetics were measured at five sequential injections of HER2 at increasing concentrations from 1.22 to 100 nM. The chip was regenerated for 30 s using 10 mM glycine (pH 1.7). A flow rate of 30 μL/min was used, and the time for adsorption and desorption was 300 and 300 s, respectively. A 1:1 binding model was used in the Biacore T200 Evaluation software version 3.1 (Cytiva, Uppsala, Sweden) to fit the data.

### Analytical Size Exclusion Chromatography (SEC) Coupled with Right-Angle Light Scattering to Study Pertuzumab Aggregation after Stressing

SEC-SLS analysis was conducted on an OMNISEC multi-detector GPC/SEC (Malvern Panalytical, Worcestershire, UK) equipped with a refractive index, right-angle light scattering (RALS), and UV/VIS diode array detector. For a detailed description of the experiment, see page 3 of the Supporting Information.

### Clinical Samples Analysis

Blood samples were collected at the Netherlands Cancer Institute (NKI, Amsterdam) from patients with stage II–III HER2-positive breast cancer who were treated with a combination of trastuzumab and pertuzumab (intravenous administration every 3 weeks of 6 mg/kg trastuzumab, with a single loading dose of 8 mg/kg, and 420 mg of pertuzumab, with a single loading dose of 840 mg). Blood was drawn into an EDTA-containing collection tube prior to each administration, and plasma was prepared by immediate centrifugation and stored at −70 °C until analysis. Enrichment of pertuzumab from clinical samples was performed, as reported previously.^22^ For a detailed description of the experiment, see page 4 of the Supporting Information.

## Results

### Cation-Exchange Profile of Stressed Pertuzumab

**Figure 1:**
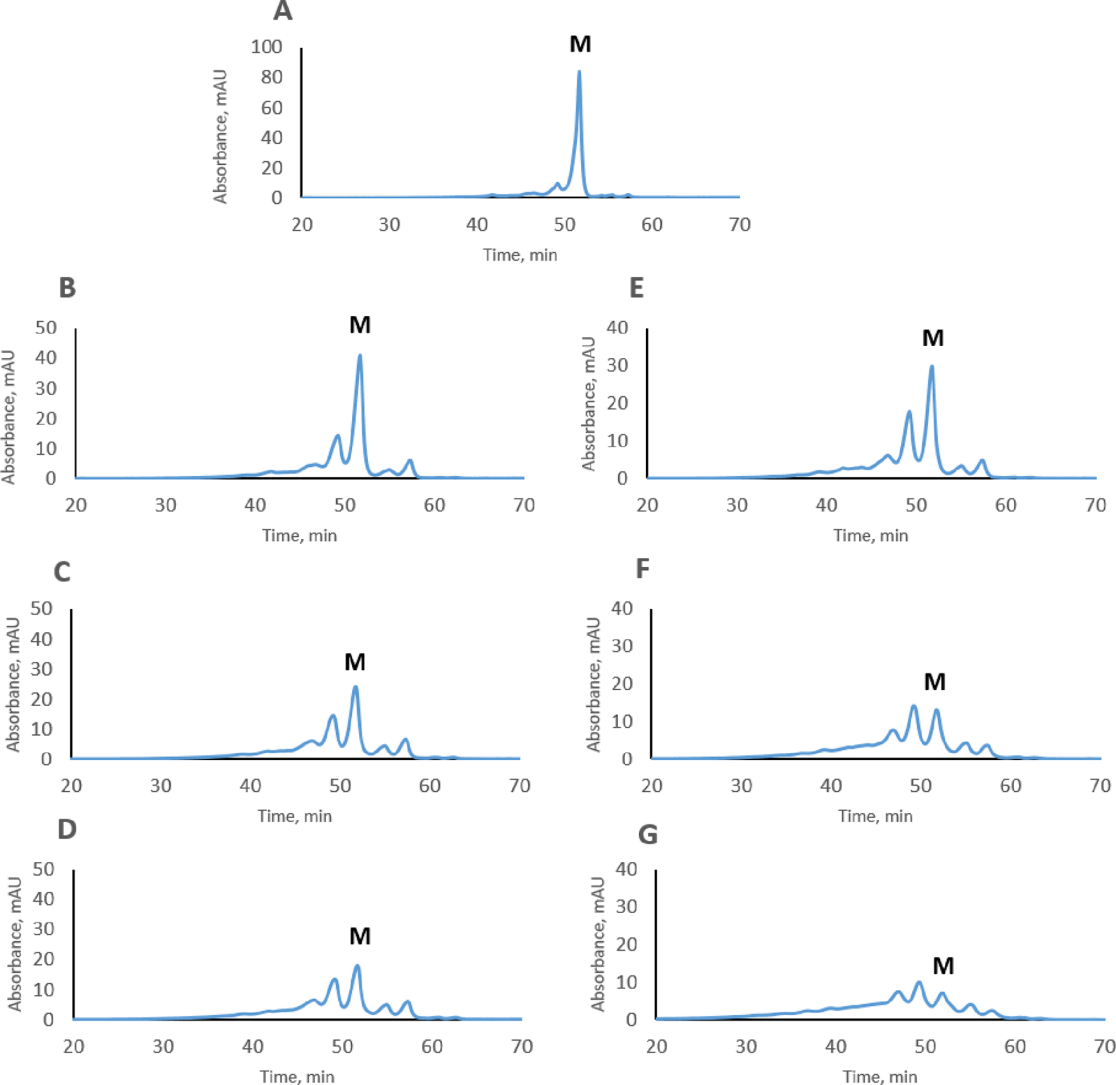
Cation-exchange profiles of non-stressed (A), stressed at 37 °C for 1, 2, and 3 weeks in PBS, pH 7.4 (B, C, D), and for 1, 2, and 3 weeks in HEPES, pH 8.5 (E, F, G), pertuzumab, respectively. “M” is an abbreviation for the main variant of pertuzumab. UV absorbance was measured at 280 nm.

The charge heterogeneity of pertuzumab stressed under different conditions is shown in Figure 1. Pertuzumab stressed in PBS (pH 7.4) and HEPES (pH 8.5) buffers showed similar profiles with degradation of pertuzumab being faster at pH 8.5. After 3 weeks of stressing, the formation of two acidic and two basic peaks was observed. These results indicate that pertuzumab is more stable than trastuzumab under the same conditions and thus less heterogeneous in terms of charge variants.

### CDR Susceptibility to Deamidation and Peptide Mapping of Stressed Pertuzumab

**Figure 2:**
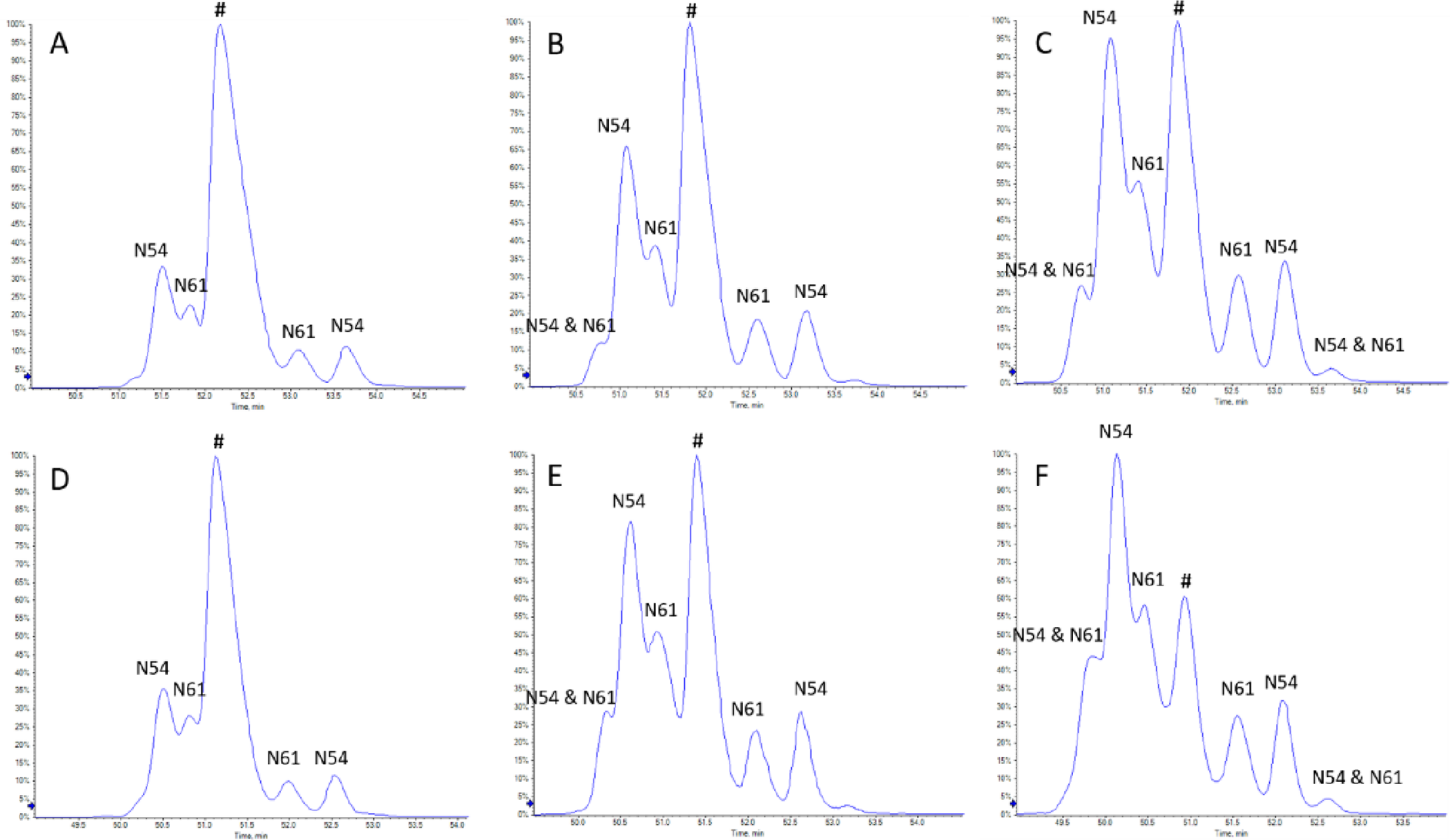
LC–MS chromatograms of the deamidation products of the heavy chain CDR2 signature peptide GLEWVADVNPN(54)SGGSIYN(61)QR* from pertuzumab acquired in a data-dependent acquisition mode. Identification of deamidation sites was performed based on the MS/MS spectra presented in Figure S2. Panels A, B, and C correspond to 1, 2, and 3 weeks in PBS (pH 7.4), and panels D, E, and F correspond to 1, 2, and 3 weeks in HEPES (pH 8.5), respectively. The native (non-deamidated) peptide is indicated with the symbol #. Deamidated peptides are indicated as either N54 or N61, depending on the deamidation position.

Successful identification of deamidation sites requires chromatographic separation of native (non-deamidated) and deamidated peptides.^14^ Therefore, the tryptic, stable-isotope-labeled (SIL) GLEWVADVNPNSGGSIYNQR* signature peptide from the heavy chain CDR2 of pertuzumab was stressed for up to 3 weeks in PBS (pH 7.4) and HEPES (pH 8.5) buffers, and these stressed samples were used for optimization of the chromatographic separation. Asparagine deamidation in positions N54 and N61 was observed when the SIL peptide was stressed (Figure 2). Identification of deamidation sites was performed based on differences in MS/MS fragmentation between native and deamidated peptides (Figure S2). Deamidation at both N54 and N61 resulted in two products for each position, which is likely due to the formation of both the Asp and isoAsp forms. When trifluoroacetic acid (TFA) is used as a mobile phase additive, isoAsp forms often elute earlier than the corresponding Asp forms in reversed-phase chromatography.^23−26^ To reduce ionization suppression, we used DFA as a mobile phase additive, which is an analogue of TFA.^27−29^ We observed a similar chromatographic behavior, and so it is likely that the earlier eluting deamidated peptides labeled N54 or N61 in Figure 2 contain an isoAsp and the later eluting peptides an Asp. Doubly deamidated peptides at N54 and N61 were also observed after 2 weeks of stressing. Deamidation at N52, which is followed by a proline (P53), was not observed.

Although the exposure of the signature peptide to stress conditions resulted in a multiproduct mixture of deamidated peptides, enzymatic digestion of stressed pertuzumab showed almost no such deamidation products. Deamidation of N54 was around 1% in pertuzumab stressed for 3 weeks in PBS and around 2% in pertuzumab stressed for 3 weeks in HEPES (Table 1).

**Table 1:** Modifications Observed in 3 Weeks’ Stressed Pertuzumab^a^

samples	Hc_N54 deamidation, %	deamidation in the Fc domain (Hc_N386 and Hc_N391), %	heavy chain N-terminal pyroGlu formation, %
3 weeks’ stressed pertuzumab in PBS	0.9 ± 0.1	18.5 ± 0.5	13.5 ± 0.1
3 weeks’ stressed pertuzumab in HEPES	1.9 ± 0.1	33.0 ± 0.3	17.3 ± 0.1

a Numbers are the average of two replicates.

Deamidation in the Fc domain and N-terminal pyroglutamate formation in the heavy chain of pertuzumab are the major modifications according to peptide mapping of the 3 week stressed samples (Table 1). Deamidation in positions N386 and N391 was detected in the Fc domain, which is a common deamidation site for mAbs.^30−32^ Samples that were stressed in HEPES buffer had a higher amount of modification compared to the samples that were stressed in PBS, which is likely due to the effect of a higher pH.^33−35^

### Characterization of Fractions from Cation-Exchange Chromatography

Intact mass measurements were performed to assess the overall differences in molecular weight between the charge variants. Modifications such as methionine oxidation, pyroglutamate formation, and C-terminal lysine truncation all result in mass changes that are measurable at the intact protein level. Subtle mass changes due to deamidation are, however, challenging to distinguish at the intact mAb level, although some progress has been reported.^36,37^ In our study, acidic variants of pertuzumab had a mass increase of about 4 Da compared to the main form (Table 2 and Figure S3), indicating that these variants may be due to deamidation of asparagine or glutamine. Basic variants had a mass decrease of around 16–17 Da, which may indicate N-terminal pyroglutamate formation in the heavy chain of the antibody.

**Table 2:** Intact Mass Measurement of Fractions Collected by Cation-Exchange Chromatography (Figure 3)^a^

fraction	measured average molecular weight for the G0F/G0F forms (Da)	mass difference compared to the main variant (Da)
M	148090.53	
A1	148094.69	+4.16
A2	148094.69	+4.16
B1	148074.59	–15.94
B2	148073.50	–17.03

a Numbers are the average of two measurements.

**Figure 3:**
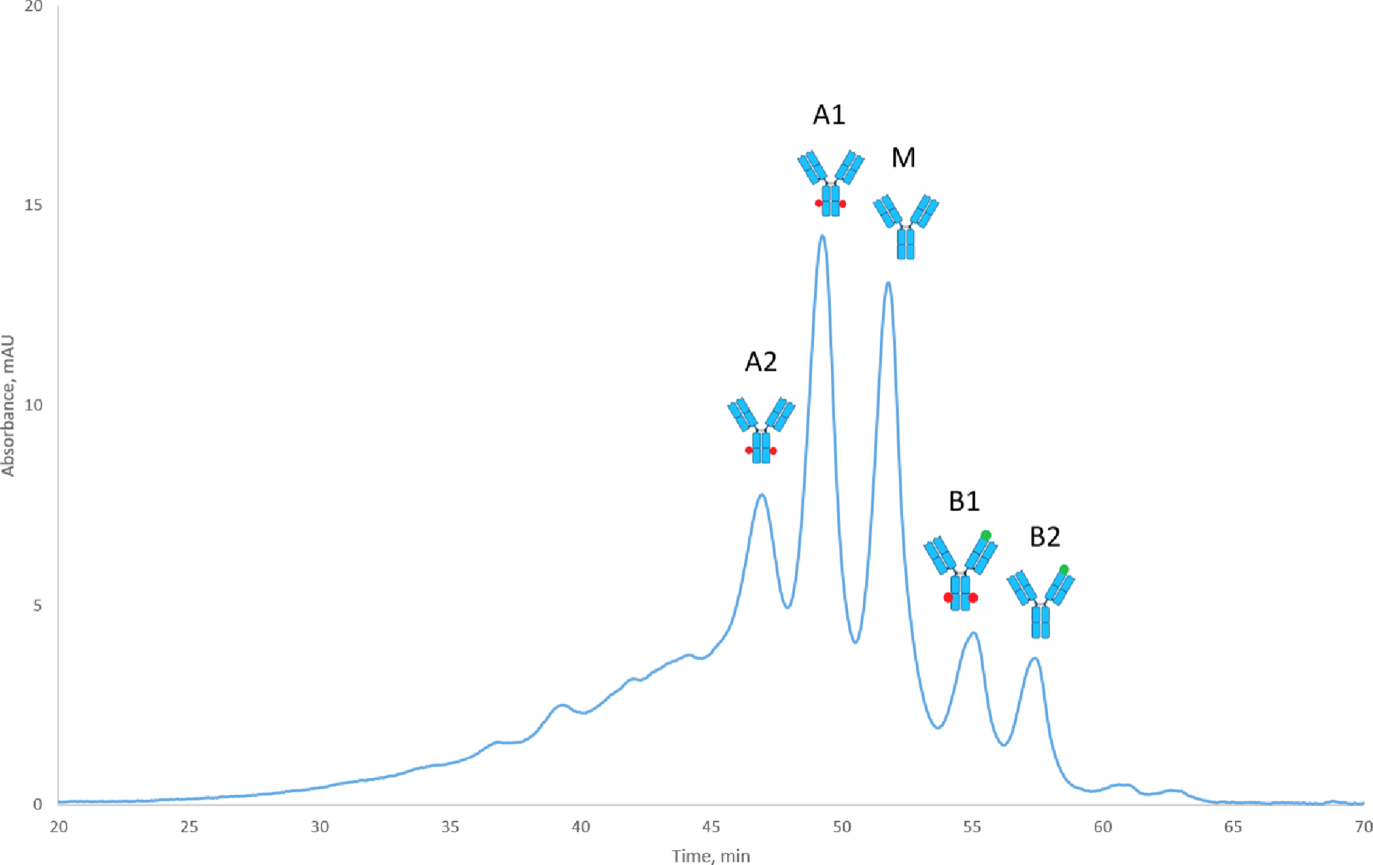
Assignment of charge variants of pertuzumab after intact mass measurements and peptide mapping. “M” is an abbreviation for the main variant, “A” is an abbreviation for acidic variants, and “B” is an abbreviation for basic variants of pertuzumab. Deamidation in the Fc domain is indicated with red dots, and N-terminal pyroGlu formation is indicated with green dots. Charge variants were collected from 2 weeks’ stressed pertuzumab in HEPES (pH 8.5) buffer. UV absorbance was measured at 280 nm.

The results of peptide mapping of the collected fractions showed that indeed both acidic forms are due to asparagine deamidation in the Fc domain of pertuzumab (Table 3 and Figure 3). The tryptic GFYPSDIAVEWESN(386)GQPEN(391)NYK peptide was used to assign deamidation in the Fc domain. Peptides with deamidations at N386 and N391 were detected in both acidic variants A1 and A2; however, the difference between the two acidic variants remained unclear. It is likely that deamidation of N386 and N391 occurred in different arms of the antibody since no peptide with double deamidation was detected. Both basic variants had a considerable amount of pyroglutamate formation, indicating that they are variants with pyroglutamate in one arm. Deamidation in the Fc domain was also found in the first basic peak explaining the slightly higher acidity of B1 compared to B2. Detection of low amounts of deamidation and pyroglutamate in the main fraction may be due to the fact that charge variants were not baseline separated on the cation-exchange column, and cross contamination from neighboring fractions could happen during fraction collection (Figure 3).

**Table 3:** Peptide Mapping Results of Fractions Collected by Cation-Exchange Chromatography (Figure 3)^a^

fraction	deamidation in the Fc domain (Hc_N386 and Hc_N391), %	heavy chain N-terminal pyroGlu formation, %
M	6%	7.8%
A1	**27.7%**	4.65%
A2	**37.8%**	6%
B1	**23.6%**	**42.9%**
B2	2.6%	**47%**

a Numbers are the average of two runs.

### SPR Analysis of Stressed Pertuzumab

**Figure 4:**
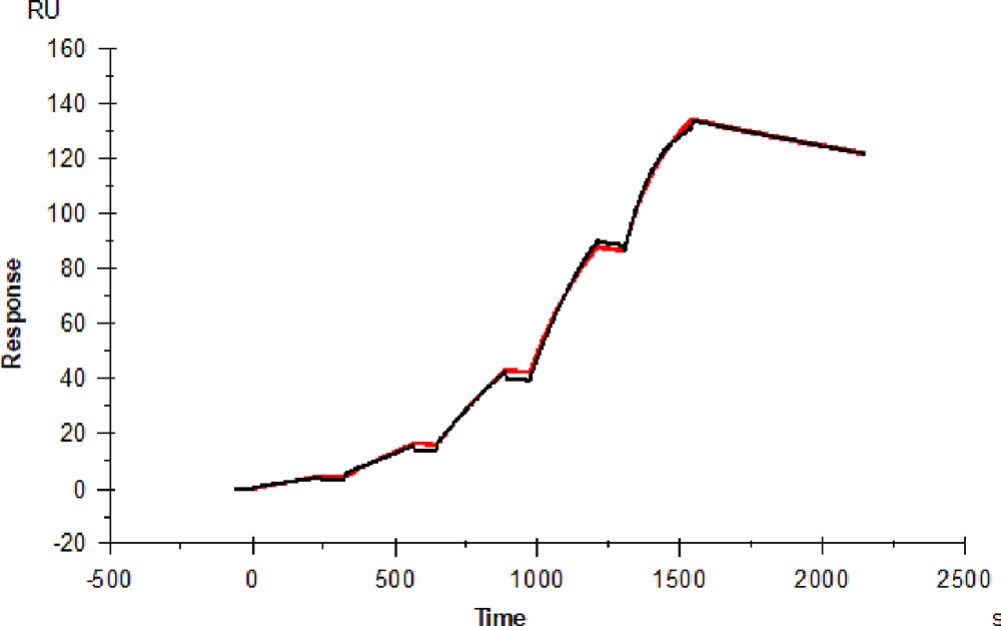
SPR single-cycle kinetics of the pertuzumab–HER2 ECD interaction. The red trace shows the measured response with increasing concentrations (from 1.22 × 10^–9^ M to 1 × 10^–7^ with three times increase at each step), and the black trace shows a 1:1 binding model that was fitted to the data. The sensorgram shows unstressed pertuzumab.

The binding of stressed and non-stressed pertuzumab to the ECD of its target antigen HER2 was measured by SPR. Since such interactions are highly influenced by the aggregation of one of the binding partners, we used SEC coupled to static light scattering as quality control, indicating very low levels of aggregation of pertuzumab upon stressing (Figure S4). Single-cycle SPR measurements were used to determine the kinetic parameters *k*_on_ and *k*_off_, as well as the dissociation constant *K*_D_, as shown in Figure 4. All samples evaluated had binding affinities of ∼2 nM, and no significant differences in binding affinity or kinetics were determined due to the observed modifications (Table 4 and Figure S5). We did not measure HER2 ECD binding for individual charge variants of pertuzumab, since modifications located in the Fc region and at the N-terminus of charge variants are not expected to affect antigen binding.^12,38,39^

**Table 4:** Pertuzumab–HER2 ECD Interaction Parameters as Determined by SPR

sample	*K*_D_ [M]	*k*_on_ [M^–1^ s^–1^]	*k*_off_ [s^–1^]
control (non-stressed)	1.8 × 10^–09^	8.6 × 10^+04^	1.6 × 10^–04^
1 week stressed in PBS, pH 7.4	2.4 × 10^–09^	6.9 × 10^+04^	1.7 × 10^–04^
2 weeks stressed in PBS, pH 7.4	2.3 × 10^–09^	7.1 × 10^+04^	1.7 × 10^–04^
3 weeks stressed in PBS, pH 7.4	2.5 × 10^–09^	6.9 × 10^+04^	1.7 × 10^–04^
1 week stressed in HEPES, pH 8.5	1.8 × 10^–09^	9.0 × 10^+04^	1.6 × 10^–04^
2 weeks stressed in HEPES, pH 8.5	1.9 × 10^–09^	8.8 × 10^+04^	1.7 × 10^–04^
3 weeks stressed in HEPES, pH 8.5	2.0 × 10^–09^	8.9 × 10^+04^	1.8 × 10^–04^

### Clinical Samples Analysis

LC–MS/MS peptide mapping analysis of plasma samples from patients treated with pertuzumab was performed after the targeted enrichment of pertuzumab and its charge variants from these samples using specific Affimers^22^ (Table 5). Plasma samples from three subjects taken at four different time points throughout their treatment were analyzed in duplicate. Patients received a fresh dose of trastuzumab and pertuzumab every 3 weeks. Blood samples were collected prior to a new dose of the mAbs was administered. Accordingly, both trastuzumab and pertuzumab circulated for 3 weeks before sample collection. The modifications that were found in clinical samples were similar to the ones from the *in vitro* stress study (Table 1). However, the level of modifications detected *in vivo* was higher than in samples stressed *in vitro* for 3 weeks under physiological conditions (stressed in PBS (pH 7.4) at 37 °C). The reason for this discrepancy may be the contribution of proteoforms from the previous injection(s) that are still circulating in the blood and that were thus exposed for longer time periods to physiological conditions. mAbs are known to have a relatively long half-life (3–4 weeks), and it is not unlikely that more highly modified proteoforms from the previous injection(s) may interfere with the quantification before they are eliminated from the body or bind to the target receptor.

**Table 5:** Peptide Mapping Results of Clinical Samples^a^

sample	Hc_N54 deamidation, %	deamidation in the Fc domain (Hc_N386 and Hc_N391), %	heavy chain N-terminal pyroGlu formation, %
**subject #135 (S135)**			
S135_TP3 (67 days)	1.8 ± 0.1	19.3 ± 0.2	9.6 ± 0.1
S135_TP4 (83 days)	2.4 ± 0.1	24.1 ± 1.2	14.0 ± 0.5
S135_TP5 (103 days)	2.7 ± 0.1	25.3 ± 0.3	14.8 ± 0.1
S135_TP6 (111 days)	3.3 ± 0.1	21.5 ± 0.6	11.7 ± 0.2
**subject #172 (S72)**			
S172_TP3 (38 days)	1.6 ± 0.2	18.1 ± 1.1	7.2 ± 0.1
S172_TP4 (59 days)	2.3 ± 0.1	23.2 ± 1.9	10.9 ± 0.3
S172_TP6 (84 days)	2.7 ± 0.2	23.4 ± 0.8	12.4 ± 0.4
S172_TP7 (104 days)	2.8 ± 0.2	24.7 ± 1.6	13.5 ± 0.2
**subject #183 (S183)**			
S183_TP2 (24 days)	1.4 ± 0.1	16.0 ± 1.1	6.9 ± 0.1
S183_TP3 (45 days)	2.3 ± 0.3	17.6 ± 0.3	9.5 ± 0.3
S183_TP4 (66 days)	2.7 ± 0.1	19.2 ± 0.3	11.0 ± 0.1
S183_TP5 (86 days)	3.0 ± 0.3	20.6 ± 2.2	12.2 ± 0.2

a Numbers are the average of two measurements.

## Discussion

We have assessed the stability of pertuzumab under physiological and basic pH conditions at 37 °C by cation-exchange chromatography. The approach using highly linear pH gradient buffers for the separation of charge variants of mAbs was used in this study.^10,21,40^ The charge variants resolved on the cation-exchange column were characterized by peptide mapping, revealing a source of charge heterogeneity associated with deamidation in the Fc domain and N-terminal pyroglutamate formation in the pertuzumab heavy chain. These modifications are quite common for the majority of antibodies and were reported earlier.^35,39,41−44^ Asparagine deamidation in the Fc domain and N-terminal pyroglutamate formation in the heavy chain are considered non-CQAs. To our knowledge, this is the first study where the stability and charge heterogeneity of pertuzumab are studied.

The susceptibility to deamidation of the pertuzumab heavy chain CDR2 under stress conditions was evaluated in this study. Although the signature tryptic peptide was susceptible to deamidation, stressed pertuzumab showed a very low level of deamidation. Similar cases have been reported in the literature where deamidation occurs in signature peptides but is many times slower or not observed at all in the corresponding protein.^42^ The higher-order structure of the protein and conformational restrictions may be the underlying reasons.

Recently, Bults et al. reported the quantification of pertuzumab by LC–MS/MS using two signature peptides, namely, FTLSVDR and GLEWVADVNPNSGGSIYNQR.^45^ Pertuzumab concentrations measured with the GLEWVADVNPNSGGSIYNQR peptide were lower than those measured with the FTLSVDR peptide. Accordingly, it was assumed that one of the asparagine residues in GLEWVADVNPNSGGSIYNQR might be deamidated, resulting in a difference in the measured concentration. However, since the authors did not analyze the deamidated peptides themselves, the difference in measured concentrations may also be due to incomplete digestion or another type of modification.

The SPR affinity measurements of stressed and non-stressed pertuzumab to HER2 ECD showed no significant changes in binding. Often, amino acid modifications in the CDRs can negatively affect the antibody–antigen interaction, such as for trastuzumab, where stressed trastuzumab, due to modifications in the CDRs, had decreased affinity to HER2 ECD.^11^ Pertuzumab is a mAb with two identical Fab domains and is expected to have bivalent binding to HER2 ECD, similar to that of trastuzumab.^46−50^ Deamidation of Asn-30 in the light chain CDR1 of trastuzumab in one of the two Fab domains resulted in a decrease in its affinity to HER2 ECD.^11^ We would thus expect to see an effect on binding by SPR in case of significant levels of deamidation in a CDR in one of the Fab domains of pertuzumab as well. However, we observed only a very small degree of deamidation in the heavy chain CDR2 of pertuzumab upon stressing, resulting in no change in binding as measured by SPR. This is in agreement with earlier studies, showing that stable CDRs and unaffected antigen binding are correlated.^51^

In this study, we have applied an anti-pertuzumab Affimer to enrich pertuzumab and its different forms from patient plasma samples.^22^ The enriched pertuzumab was analyzed by peptide mapping after enzymatic digestion. The results of the analysis of patient samples indicated that *in vivo* biotransformation can be mimicked by stressing *in vitro*. These findings could be useful for designing new antibodies and scaffolds.

## Supporting Information Available

Additional experimental details and supporting figures showing amino acid sequence of pertuzumab (Figure S1); LC–MS/MS spectrum of native and deamidated peptides (Figure S2); intact mass spectrum of fractions (Figure S3); SEC-RALS of unstressed and stressed pertuzumab (Figure S4); and SPR sensorgrams of stressed pertuzumab (Figure S5) (PDF)

## Notes

The clinical study including sample collection was approved by the medical ethics committee of the Netherlands Cancer Institute. All patients provided written informed consent prior to study inclusion and separately for sample collection. All procedures performed in this study involving human participants were in accordance with the ICH Harmonized Tripartite Guideline for Good Clinical Practice and with the 1964 Helsinki Declaration and its later amendments.
