# Supplementary material for: Pertuzumab Charge Variant Analysis and Complementarity-Determining Region Stability Assessment to Deamidation
Source: Anal Chem. 2023 Feb 16;95(8):3951–8. doi: 10.1021/acs.analchem.2c03275 (PMC9979147; doi:10.1021/acs.analchem.2c03275)
Supplement: Supplementary file 1 — ac2c03275_si_001.pdf [file ac2c03275_si_001.pdf]

Supporting Information

Pertuzumab charge variant analysis and CDR stability assessment to deamidation

Baubek Spanov[a], Oladapo Olaleye[a], Tomás Mesurado[b], Natalia Govorukhina[a], Alois Jungbauer[b], Nico C. van de Merbel[a, c], Nico Lingg[b], Rainer Bischoff[a*]

[a] *Department of Analytical Biochemistry, Groningen Research Institute of Pharmacy, University of Groningen, A Deusinglaan 1, 9713 AV Groningen, The Netherlands*
[b] *Department of Biotechnology, Institute of Bioprocess Science and Engineering, University of Natural Resources and Life Sciences, Vienna, Muthgasse 18, Vienna 1190, Austria*
[c] *Bioanalytical Laboratory, ICON, Amerikaweg 18, 9407 TK Assen, The Netherlands*

Corresponding Author: Rainer Bischoff − *Department of Analytical Biochemistry, Groningen Research Institute of Pharmacy, University of Groningen, 9713 AV Groningen, The Netherlands; orcid.org/0000-0001-9849-0121; Email: r.p.h.bischoff@rug.nl*

Table of content

	Title	Page #
**Experimental protocol**	MS settings for LC-MS/MS peptide mapping	**3**
**Experimental protocol**	Intact mass measurement parameters	**3**
**Experimental protocol**	Size-exclusion chromatography	**3**
**Experimental protocol**	Enrichment protocol	**4**
**Figure S1**	Pertuzumab amino acid sequence	**5**
**Figure S2**	LC-MS/MS spectra of native and deamidated peptides	**5**
**Figure S3**	Mass spectrum of fractions	**6**
**Figure S4**	SEC-RALS of unstressed and stressed pertuzumab	**7**
**Figure S5**	SPR sensorgrams of stressed pertuzumab	**7**
**References**	The reference list	**8**

Experimental protocols

LC-MS/MS peptide mapping. An Eksigent NanoLC 425 system with a microflow pump (1-10 µL) coupled to a TT6600 quadrupole-time-of-flight (QTOF) mass spectrometer with an OptiFlow® source (SCIEX, Toronto, Canada) was used for LC-MS/MS peptide mapping. The following source settings were used: Ion Source Gas 1 (GS1) 10 psi, Ion Source Gas 2 (GS2) 20 psi, Curtain Gas (CUR) 25 psi, Temperature (TEM) 100 °C, IonSpray Voltage Floating (ISVF) 4500 V, and Declustering Potential (DP) 90 V. The data-dependent acquisition mode was used for MS/MS analyses where one cycle consisted of an MS scan from 350 to 2000 m/z, followed by MS/MS of the top ten most intense precursor ions detected at a minimum threshold of 100 counts per second. MS/MS fragmentation was performed on precursor ions with a charge state of 2 to 4 with an exclusion window of 6 seconds after two occurrences. Data analysis was done with the BPV Flex 2.1 and PeakView 2.2 software (SCIEX, Toronto, Canada). 15 ppm was set as precursor mass error tolerance and 0.03 Da was set as fragment mass error tolerance. Methionine oxidation and asparagine deamidation were set as variable modifications for the database (pertuzumab sequence) search.

Intact mass measurement of charge variants by Reversed-Phase LC-MS. A HALO® Diphenyl column (2.7 µm, 2.1 × 100 mm, 1000Å, Advanced Materials Technology, Delaware, USA) was used for reversed-phase LC-MS. Mobile phase A contained 0.05% DFA in water and mobile phase B contained 0.05% DFA in acetonitrile. A gradient from 10 to 50% B was applied at a flow rate of 0.4 mL/min. The column was heated to 80 °C. The positive ion mode was used for MS spectra acquisition with the following source settings: nebulizer gas 3 bar; dry gas rate 12 L/min; dry gas temperature 250 °C; capillary voltage 4500 V; funnel RF 400 Vpp; multipole RF 400 Vpp; ion energy 4.0 eV; isCID 120 eV; collision energy, 8.0 eV; collision RF 3000 Vpp.

Analytical Size Exclusion Chromatography (SEC) coupled with right-angle light scattering to study pertuzumab aggregation after stressing. Stressed pertuzumab samples were separated on a Superdex S200 increase 10/300 GL column (Cytiva, Upsala, Sweden) maintained at 25 °C, using PBS as an isocratic mobile phase at a flow rate of 0.5 mL/min. The autosampler chamber was maintained at 25 °C. The injection volume varied between 20 and 100 µL. The detectors were maintained at 25 °C. Protein concentration was measured online by using the refractive index detector. A dn/dc of 0.185 was taken. The instrument was calibrated using commercially available BSA (2 mg/mL) (Thermo Scientific™ Pierce™). Sample was not filtered or centrifuged prior to analysis. Since the relative mass contribution of glycans (around 1–3%) in antibodies is rather low, the impact on dn/dc was neglected.[1]

Clinical samples analysis. Enrichment of pertuzumab from clinical samples was performed as reported previously.[2] Briefly, 12 µg anti-pertuzumab affimer 709213 (Avacta Life Sciences, Wetherby, United Kingdom) was coupled to 40 µL of maleimide beads overnight at pH 9 and room temperature. The next day, beads were first washed with carbonate buffer pH 9 and then blocked with 50 mM cysteine for 1 hour in the dark. Afterwards, beads were washed three times with Tris buffer pH 8 and three times with PBS pH 7.4 and transferred to a Protein LoBind 96 well plate. Enrichment was performed by adding 50 µL of PBS and 50 µL of plasma samples per well, followed by incubation for 1.5 hours. After enrichment, the beads were washed three times with PBS containing 0.05% Tween-20 and three times with PBS alone to remove excess Tween-20. On-bead digestion of enriched pertuzumab involved the following two steps: first, 40 µL of 0.3% SDC in 50 mM HEPES pH 7 buffer and 10 µL of 50 mM DTT were added per well and incubated for 30 min at 60 °C. Afterwards, 5 µL of 0.1 µg/µL Trypsin/Lys-C mix was added, and proteins digested for 6 hours at 37 °C. SDC was removed by precipitation with 0.4% DFA and centrifugation for 10 min at 10 000 rpm. 5 µL of the digest was injected for the LC-MS analysis.

Supplementary figures:

>Amino acid sequence for pertuzumab light chain

DIQMTQSPSSLSASVGDRVTITC**KASQDVSIGVA**WYQQKPGKAPKLLIYS**ASYRYT**GVPS
RFSGSGSGTDFTLTISSLQPEDFATYYC**QQYYIYPYT**FGQGTKVEIKRTVAAPSVFIFPP
SDEQLKSGTASVVCLLNNFYPREAKVQWKVDNALQSGNSQESVTEQDSKDSTYSLSSTLT
LSKADYEKHKVYACEVTHQGLSSPVTKSFNRGEC

>Amino acid sequence for pertuzumab heavy chain

EVQLVESGGGLVQPGGSLRLSCAAS**GFTFTDYTMD**WVRQAPGKGLEWVA**DVNPNSGGSIY**
NQRFKGRFTLSVDRSKNTLYLQMNSLRAEDTAVYYCARN**LGPSFYFDY**WGQGTLVTVSSA
STKGPSVFPLAPSSKSTSGGTAALGCLVKDYFPEPVTVSWNSGALTSGVHTFPAVLQSSG
LYSLSSVVTVPSSSLGTQTYICNVNHKPSNTKVDKKVEPKSCDKTHTCPPCPAPELLGGP
SVFLFPPKPKDTLMISRTPEVTCVVVDVSHEDPEVKFNWYVDGVEVHNAKTKPREEQYNS
TYRVVSVLTVLHQDWLNGKEYKCKVSNKALPAPIEKTISKAKGQPREPQVYTLPPSREEM
TKNQVSLTCLVKGFYPSDIAVEWESNGQPENNYKTTPPVLDSDGSFFLYSKLTVDKSRWQ
QGNVFSCSVMHEALHNHYTQKSLSLSPG

Figure S1. Amino acid sequence of pertuzumab (https://go.drugbank.com/drugs/ DB06366). CDR amino acids are shown in bold.

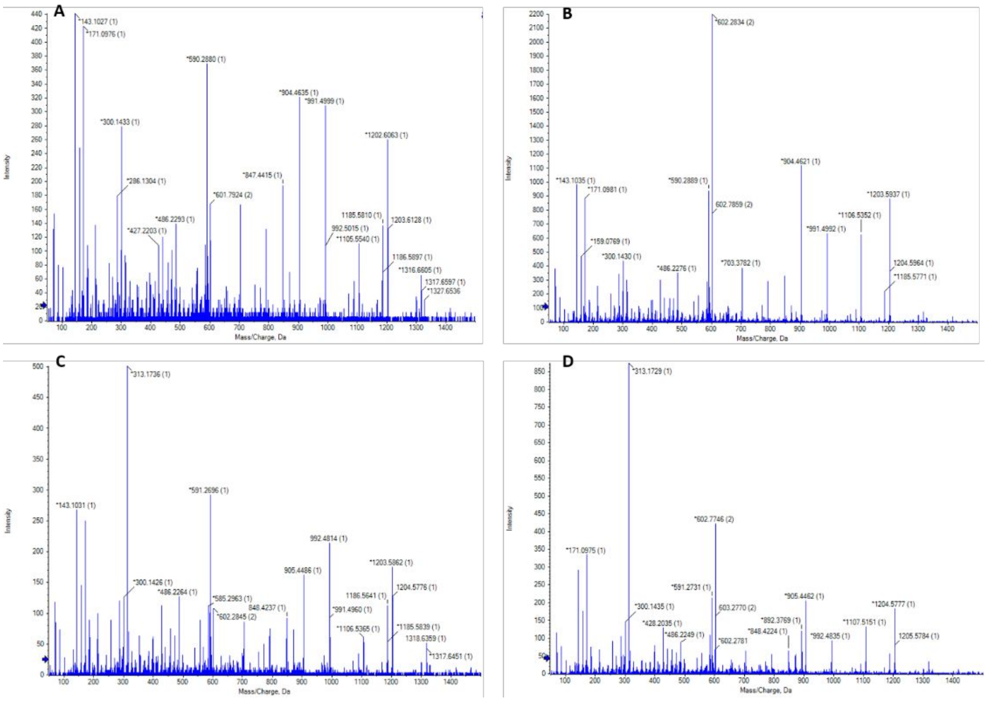

Figure S2. LC-MS/MS spectra of (A) native (non-deamidated), (B) deamidated at N54, (C) deamidated at N61, and (D) deamidated at N54 and N61 GLEWVADVNPNSGGSIYNQR* SIL peptide shown in Figure 2.

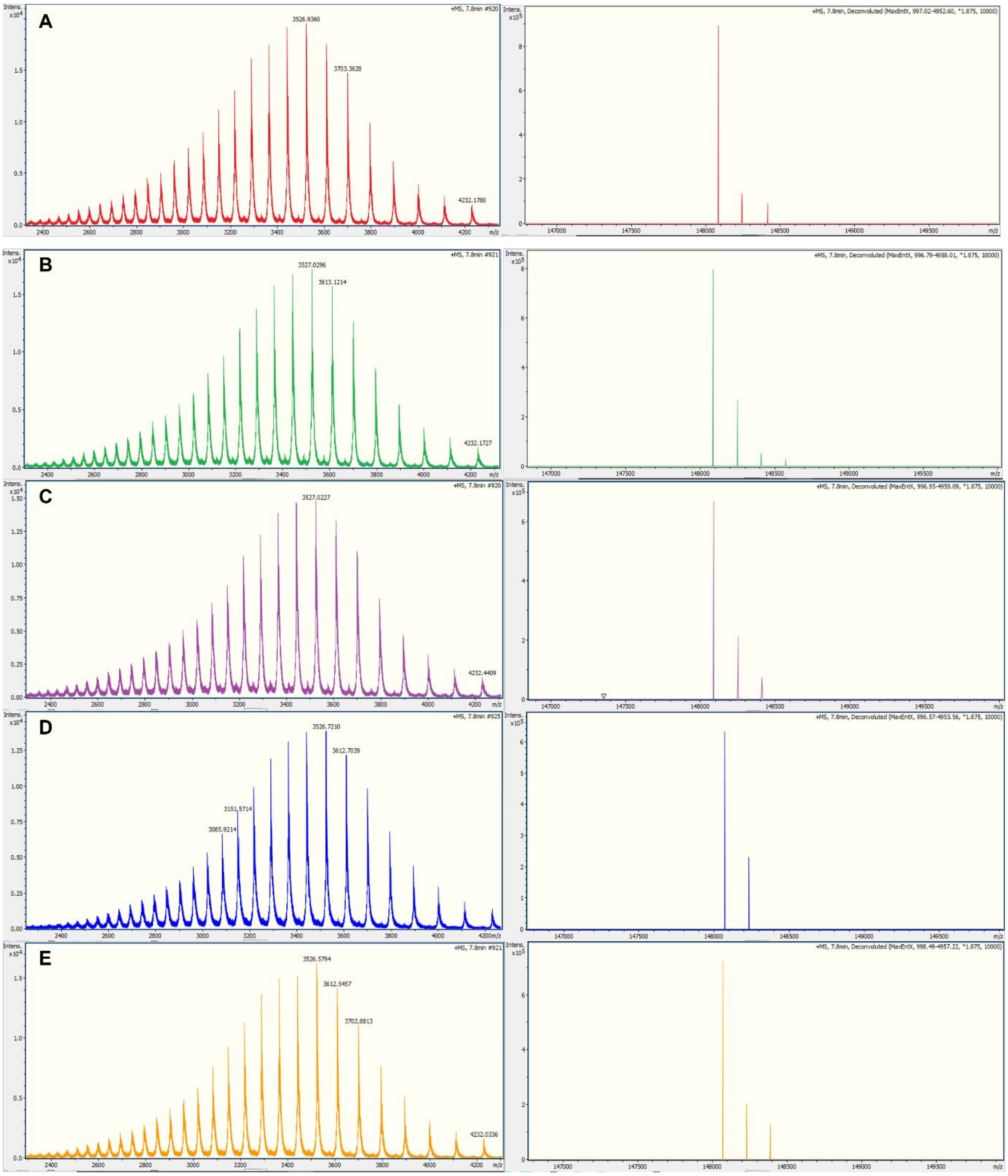

Figure S3. Raw (left) and deconvoluted (right) mass spectra obtained by RPLC-MS on a Maxis Plus QTOF instrument (Bruker). (A) Main fraction; (B) fraction A1; (C) fraction A2; (D) fraction B1; (E) fraction B2 from **Figure 3** and **Table 3**

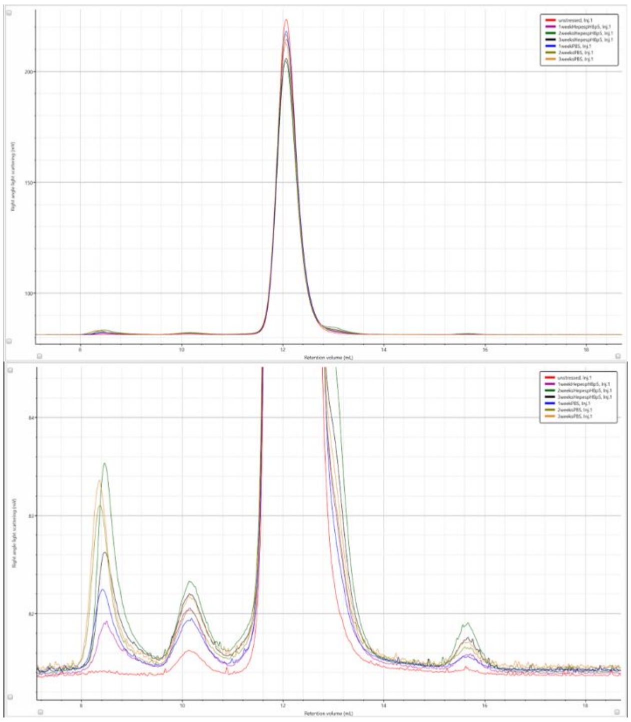

Figure S4. SEC-RALS of unstressed and stressed pertuzumab. The right-angle light scattering signal is shown. A very small increase of dimers and multimers was noticed in the stressed samples. Furthermore, a slight shoulder was evident in the stressed samples, in particular week 2 and week 3 at pH 8.5, where the mass of the shoulder was determined to be 103 – 111 kDa.

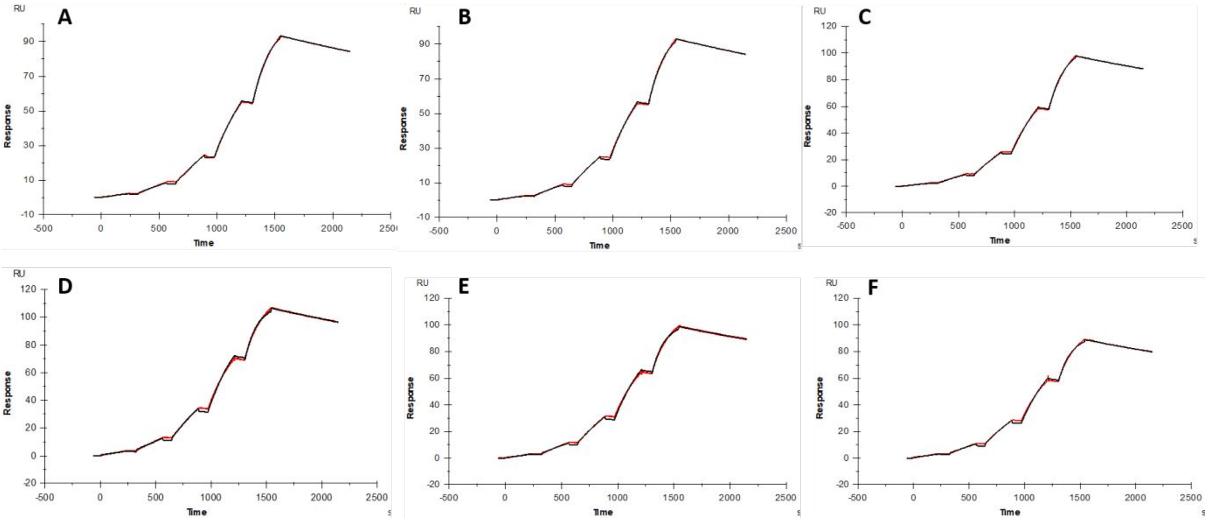

Figure S-5. SPR single cycle kinetics of the pertuzumab-HER2 ECD interaction. The red trace shows the measured response with increasing concentrations (from 1.22E-9M to 1E-7 with 3 times increase at each step) and the black trace shows a 1:1 binding model that was fitted to the data. Sensograms shown here correspond to stressed pertuzumab (A) 1 week in PBS pH 7.4, (B) 2 weeks in PBS pH 7.4, (C) 3 weeks in PBS pH 7.4, (D) 1 weeks in HEPES pH 8.5, (E) 2 weeks in HEPES pH 8.5, (F) 3 weeks in HEPES pH 8.5

References:

(1) Hoffmann, A.; Grassl, K.; Gommert, J.; Schlesak, C.; Bepperling, A. Precise Determination of Protein Extinction Coefficients under Native and Denaturing Conditions Using SV-AUC. *Eur. Biophys. J.* **2018**, *47* (7), 761–768. https://doi.org/10.1007/s00249-018-1299-x.

(2) Olaleye, O.; Spanov, B.; Ford, R.; Govorukhina, N.; van de Merbel, N. C.; Bischoff, R. Enrichment and Liquid Chromatography-Mass Spectrometry Analysis of Trastuzumab and Pertuzumab Using Affimer Reagents. *Anal. Chem.* **2021**, *93* (40), 13597–13605. https://doi.org/10.1021/acs.analchem.1c02807.
